# New Physiological Journal by Brown-Séquard

**Published:** 1858-01

**Authors:** 


WE have received the prospectus of a new journal about being commenced in Paris, under the editorial supervision of Dr. E. Brown-Séquard, entitled "*Journal de Physiologie de l'Homme et des Animaux.*" It is surprising that this should be the first journal published in France whose pages are devoted solely to this interesting and progressive branch of medical science; and Dr. Brown-Séquard's zeal and industry, and his world-wide reputation as a physiologist, will be a sufficient guarantee of the value of this journal. He will be assisted in his editorial labors by Drs. Ch. Robin, Ch. Rouget, and Tholozan, with the promise of full and original communications from many distinguished physiologists in Germany, England, and America.

This journal will treat, besides pure physiology, of—

1. Organic Chemistry, hygiene, toxicology, and legal medicine, in their relations to physiology.
2. Descriptive anatomy, comparative anatomy, teratology, and normal and pathological histology, so far as they may be connected with physiology.
3. The application of physiology to the practice of medicine, surgery, and obstetrics.

The numbers will be issued four times a year — on the first day of January, April, July, and October—each number containing about 200 pages.

The contents will be classified in the following manner:—

1. Original communications, forming more than one-half of each number.
2. Articles published abroad, entire or in part.

3. Critical analyses of books published in France and other countries.

4. Accounts of the progress of physiology in France and other countries.

The subscription price in this country will be twenty-five francs, or $5 per year.

American authors who wish to have their works reviewed, or those sending original communications, will please address, *post-paid*, M. J. B. Baillière et Fils, or Dr. Brown-Séquard, Rue du Dragon 16, à Paris.

---

CHANGES IN MEDICAL JOURNALS.—The proprietorship of the *New Orleans Medical and Surgical Journal* has passed into the hands of Drs. W. Stone, J. Jones, and S. Chaillé, who have also become assistant editors, Dr. Bennett Dowler retaining the position of editor-in-chief.

The *Medical News and Hospital Gazette* has also undergone a change in its management, and is now edited by Drs. D. W. Brickell and E. D. Fenner. To the latter, we believe, belongs the credit of having been the first to establish a medical periodical in the Southern Metropolis.

Dr. A. P. Merrill has resigned the charge of the *Memphis Medical Recorder* into the hands of Samuel F. Wright, M.D.

---

BOYLSTON PRIZE ESSAYS.—The premium for the best essay on "The Pathology and Treatment of Diseases of the Urinary Organs," has been awarded to Dr. W. W. Morland, of Boston; and that for the best elucidation of the question, "Under what circumstances do the usual signs furnished by auscultation and percussion prove fallacious?" to Dr. E. Cutter, of Woburn, Mass. Considering the high standing of the two successful competitors, we anticipate much pleasure and profit in the perusal of their respective dissertations.

